# Quantitative evaluation of Kalayanak Ghrita and Sarasvat Ghrita dosage using a *Drosophila* life-history framework

**DOI:** 10.17912/micropub.biology.002051

**Published:** 2026-03-30

**Authors:** Madhu G. Tapadia, Saurabh Chand Sagar

**Affiliations:** 1 Department of Zoology, Banaras Hindu University, Varanasi, Uttar Pradesh, India

## Abstract

Ayurveda, an ancient Indian system of medicine, is recognized for its holistic therapeutic approach; however, many supplementations lack experimental, dose-defined validation. Here, we examine the biological impact and optimal dosage of Ayurvedic Rasayana, Kalyanaka Ghrita and Sarasvata Ghrita, using *Drosophila melanogaster* as model organism. By analysing key life-history traits—development, fecundity, and longevity—we show that neither supplementation affects development, while higher concentrations negatively influence survival and reproductive output. Lower doses are largely well tolerated, defining a safe physiological window. This study provides quantitative insight into Ayurvedic dosage effects and demonstrates the utility of *Drosophila* for physiological evaluation of traditional medicines.

**Figure 1. The effect of the KG and SG on the longevity and fecundity pattern in wild type Drosophila melanogaster.** ***(A)* Kaplan–Meier survival analysis of *Drosophila melanogaster* fed with KG.** Log-rank (Mantel–Cox) testing revealed significant differences in lifespan among treatment groups (χ² = 17.65, df = 4, p = 0.0014), with a significant dose-dependent trend (χ² = 16.04, df = 1, p < 0.0001). Gehan–Breslow–Wilcoxon analysis indicated increased early mortality at higher KG concentrations (χ² = 17.46, df = 4, p = 0.0016). KG 1.0% significantly reduced lifespan, whereas lower concentrations showed no adverse effects ( *n* = 500 per group) *and* ***(B) Median life span*** *of the wild type Drosophila upon feeding on Normal food (NF), KG 0.10%, KG 0.25%, KG 0.50% and KG 1.0% supplementation food (n=500).* ***(C)* Kaplan–Meier survival analysis of *Drosophila melanogaster* fed with SG.** Log-rank (Mantel–Cox) testing revealed highly significant differences in lifespan among treatment groups (χ² = 67.88, df = 4, p < 0.0001), with a pronounced dose-dependent decline in survival confirmed by the log-rank test for trend (χ² = 59.98, df = 1, p < 0.0001). Gehan–Breslow–Wilcoxon analysis indicated significantly increased early mortality at higher SG concentrations (χ² = 48.71, df = 4, p < 0.0001). Lower concentrations showed minimal effects on lifespan, whereas higher SG concentrations caused a marked reduction in survival ( *n* = 500 per group) *and* ***(D) Median life span*** *of the wild type Drosophila upon feeding on Normal food (NF), SG 0.10%, SG 0.25%, SG 0.50% and SG 1.0% supplementation food (n=500).* ***(E) Day-wise effects of KG on fecundity in wild-type Drosophila melanogaster.*** Egg-laying was quantified daily from Days 4–23 post-eclosion and compared with controls using Dunnett’s multiple comparisons test. Lower concentrations (KG 0.10% and 0.25%) showed minimal or transient effects, whereas higher concentrations (KG 0.50% and KG 1.0%) significantly altered fecundity, particularly during the peak reproductive window (Days 7–10). KG 1.0% exhibited persistent reductions in egg-laying across multiple time points, indicating a dose- and age-dependent effect on reproductive output. Data are presented as mean ± SEM (n = 20 pairs per group). *p < 0.05; **p < 0.01; ***p < 0.001; ***p < 0.0001 ***. (F) Effect of SG on age-dependent fecundity in Drosophila melanogaster.*** Day-wise egg-laying output of adult female flies fed with SG at 0.10%, 0.25%, 0.50%, and 1.0% concentrations was quantified from Day 4 to Day 24 post-eclosion and compared with untreated controls. Statistical analysis was performed using Dunnett’s multiple comparisons test to evaluate treatment effects relative to control at each time point. SG supplementation resulted in a dose- and age-dependent reduction in fecundity, with SG 1.0% showing persistent and significant suppression of egg laying across most reproductive days, while lower concentrations exhibited intermittent or non-significant effects, particularly during early reproductive stages. Data are presented as mean ± SEM (n = 20 per group). Significance levels are indicated as p < 0.05 (*), p < 0.01 (**), p < 0.001 (***), and p < 0.0001 (****).

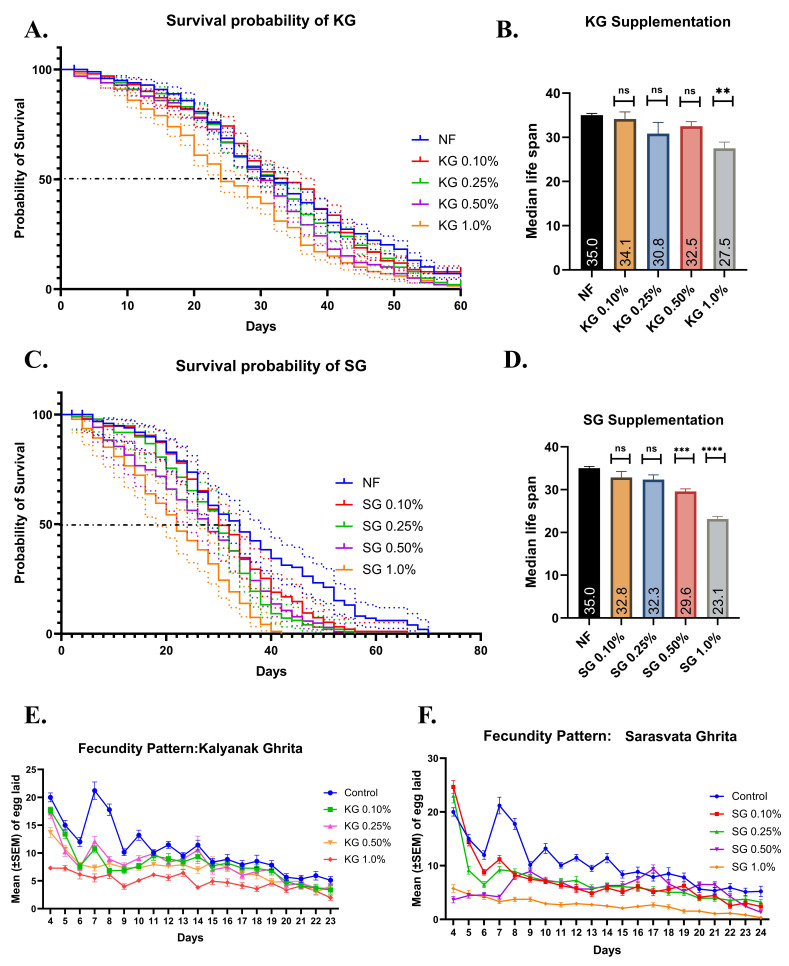



## Description

Ayurveda is a traditional system of medicine that adopts a holistic approach to health, emphasizing the treatment of disease as well as its prevention. Kalyanaka Ghrita (KG) and Sarasvata Ghrita (SG) are well-known Ayurvedic Rasayana supplementations belonging to the Medhya-Kamya Rasayana (Kaushik et al., 2021), widely prescribed for neurological disorders (Singh et al., 2023) and reported to improve cognition (Ramana et al., 2019), memory loss, Alzheimer’s disease (Badal et al., 2022), and Huntington’s disease (Sharma et al., 2021). To investigate the biological effects these supplementations, we employed *Drosophila melanogaster* as a model organism. We assessed the impact of KG and SG by examining three fundamental life-history traits: development, fecundity, and longevity. Our results indicate that neither supplementation alters developmental progression upto 1.0% formulation concentration. Both KG and SG supplementation show no effects on the fecundity pattern except during peak reproductive phase (days 7-10) at lower concentrations, however, at higher concentration both supplementations exert a detrimental effect on reproductive output. Neither supplementation improves survival under standard laboratory conditions, and at higher concentrations both KG and SG negatively affect longevity, likely reflecting dose-dependent toxicity.

Life-history traits describe the patterns of growth, reproduction, and survival across an organism’s lifespan and include parameters such as developmental timing, reproductive maturity, fertility, aging rate, and lifespan (Hillesheim and Stearns, 1992). In this study, we focused on three core life-history parameters—development, fecundity, and longevity—to evaluate effects of KG and SG. To assess dose-dependent responses, both supplementations were administered at four concentrations: 0.10%, 0.25%, 0.50%, and 1.0%; dye mixed food media was used to confirm the ingestion of Ayurvedic Rasayana supplemented food (supplementary image S1).

We performed longevity assays to assess fly survival and to identify supplementation concentrations that do not adversely affect normal lifespan. The results showed that KG supplementation up to 0.50% did not significantly alter the longevity pattern of wild-type *Drosophila melanogaster* , whereas KG 1.0% was toxic and led to a marked reduction in lifespan (n = 500 per supplementation concentration). In contrast, SG supplementation at 0.10% had no effect on longevity; however, higher concentrations—SG 0.25%, SG 0.50%, and SG 1.0%—caused a significant, dose-dependent decline in lifespan (n = 500 per supplementation concentration).

The median lifespan of wild-type *Drosophila melanogaster* was approximately 35 days; similar to the previous report from our lab (Sagar and Tapadia, 2026). This value changed to 34, 31, 32, and 27 days following supplementation with KG 0.10%, KG 0.25%, KG 0.50%, and KG 1.0%, respectively. In contrast, SG supplementation resulted in median lifespans of 33, 32, 29, and 23 days at SG 0.10%, SG 0.25%, SG 0.50%, and SG 1.0%, respectively.

Kaplan–Meier survival analysis followed by log-rank (Mantel–Cox) test revealed significant differences in lifespan among treatment groups fed on KG (χ² = 17.65, df = 4, p = 0.0014). A log-rank test for trend further demonstrated a strong dose-dependent effect on survival fed on KG (χ² = 16.04, df = 1, p < 0.0001), while Gehan–Breslow–Wilcoxon analysis indicated that early mortality significantly contributed to the reduced lifespan observed at higher supplementation concentrations (χ² = 17.46, df = 4, p = 0.0016). This observation suggests KG 1.0% supplementation feeding negatively affects the longevity, whereas, lower concentration has no effect on longevity.

Similarly, Kaplan–Meier survival analysis followed by log-rank (Mantel–Cox) testing revealed highly significant differences in lifespan among treatment groups fed on SG (χ² = 67.88, df = 4, p < 0.0001). A log-rank test for trend confirmed a strong dose-dependent effect on survival fed on SG (χ² = 59.98, df = 1, p < 0.0001), while Gehan–Breslow–Wilcoxon analysis indicated that early mortality significantly contributed to the reduced lifespan observed at higher supplementation concentrations (χ² = 48.71, df = 4, p < 0.0001).

These results indicate that SG&nbsp;0.10% and SG 0.25% supplementation concentration&nbsp;has no&nbsp;negative impact&nbsp;on the longevity pattern&nbsp;and median life span of *Drosophila* *melanogaster* ;&nbsp;however, higher&nbsp;concentrations, SG 0.50% and SG 1.0%&nbsp;supplementation&nbsp;feeding, have an adverse&nbsp;effect on&nbsp;their&nbsp;longevity&nbsp;pattern. Based upon longevity experiments it was concluded, supplementation concentration of KG 1.0% and SG 0.50% or higher proves to be toxic to the flies and reduced the longevity significantly, however, the lower concentrations of both the supplementations ( *viz.,* ≤ 1.0% KG and ≤0.50% SG supplementation concentration) show no significant change in longevity and thus are fit for oral supplementation.

Development is a key life-history trait; therefore, we next assessed whether supplementation with the Ayurvedic Rasayana supplementations affected normal developmental progression in *Drosophila melanogaster* . Analysis of developmental timing following KG supplementation revealed that approximately 99% of larvae reached the early pupal stage at 120 ± 2 hours, and nearly all successfully eclosed by the fifth day after pupation (n = 500 per supplementation concentration). Comparable results were obtained with SG supplementation, where ~99% of larvae entered the early pupal stage at 120 ± 2 hours and eclosed by the fifth day post-pupation (n = 500 per supplementation concentration). These findings indicate that neither supplementation, irrespective of concentration, perturbs normal developmental progression up to 1.0% supplementation.

Next, we performed fecundity assay as a measure of the fly fertility which is one of the three major life history traits. Upon analysing the fecundity pattern for 3 weeks after fly eclosion upon KG supplementation feeding on suggested non-significant change of 5.8% and 6.1% in average egg laying upon KG 0.1% and KG 0.25% supplementation feeding, however, higher concentration of KG 0.50% and KG 1.0% resulted in significant decrease of the 20.7% and 60.3% in the average egg laying in *Drosophila melanogaster* respectively. On the other hand, fecundity assay upon SG supplementation feeding revealed non-significant decrease of the 19.8%, 21.5%, 19.5% in the average egg laying upon SG 0.10%, SG 0.25%, SG 0.50% supplementation feeding respectively but supplementation feeding of SG 1.0% shows significant decrease of 58.89% in the average egg laying in the *Drosophila melanogaster.*

Day-wise fecundity analysis using Dunnett’s multiple comparisons test revealed a dose- and age-dependent effect of KG relative to controls. During early reproductive stages (Days 4–6), lower concentrations (KG 0.10% and KG 0.25%) generally showed no significant differences, whereas higher concentrations (KG 0.50% and KG 1.0%) exhibited significant alterations in egg-laying output. Across the peak reproductive window (Days 7–10), KG supplementation resulted in significant deviations from control fecundity, with the strongest effects observed at KG 0.50% and KG 1.0% (adjusted *p* < 0.001). During mid to late reproductive stages (Days 11–23), the effects became increasingly concentration dependent, with KG 1.0% showing persistent and significant changes in fecundity across multiple time points, while lower concentrations displayed largely non-significant differences. Collectively, these findings demonstrate that KG modulates fecundity in a dose- and age-dependent manner, with higher concentrations exerting sustained negative effects on reproductive output.

Day-wise fecundity analysis using Dunnett’s multiple comparisons test revealed a dose- and age-dependent effect of SG relative to controls. During early reproductive stages (Days 4–6), SG 0.10% generally showed no significant differences, whereas higher concentrations (SG 0.25%, SG 0.50%, and SG 1.0%) exhibited significant reductions in egg-laying output, with the strongest effects observed at SG 0.50% and SG 1.0%. Across the peak reproductive window (Days 7–10), all SG concentrations differed significantly from controls, indicating a pronounced suppression of fecundity, which intensified with increasing dose (adjusted *p* < 0.001 for most comparisons). During mid to late reproductive stages (Days 11–24), the effects became increasingly concentration dependent, with SG 1.0% showing persistent and significant reductions in fecundity across nearly all time points, while lower concentrations displayed sporadic or non-significant differences. Collectively, these results demonstrate that SG exerts a strong, dose- and age-dependent negative effect on fecundity, with higher concentrations causing sustained impairment of reproductive output.

Taken together, our study demonstrates that the Ayurvedic Rasayana supplementations KG and SG exert dose- and age-dependent effects on life-history traits in *Drosophila melanogaster* , while largely preserving normal development at all tested concentrations. Neither supplementation interfered with larval development or metamorphosis, indicating that early growth and differentiation are resilient to Rasayana supplementation. Longevity analyses revealed that both supplementations possess a restricted safe dosage window, with KG showing toxicity only at 1.0%, whereas SG exhibited adverse effects at comparatively lower concentrations (≥0.50%). Survival curve analyses further indicated that lifespan reduction at higher doses was driven in part by early adult mortality, suggesting dose-dependent physiological stress rather than gradual aging acceleration.

Reproductive fitness emerged as the most sensitive life-history trait affected by both supplementations. At lower concentrations, KG and SG largely did not alter overall fecundity, except for transient effects during the peak reproductive phase (Days 7–10). However, higher concentrations of both supplementations consistently exerted detrimental effects on reproductive output, with SG producing a more pronounced and sustained suppression of fecundity compared to KG. Day-wise analyses revealed that these effects intensified with advancing age, particularly at the higher concentrations, highlighting a strong interaction between dose, reproductive stage, and aging. Thus, it is safe to use KG 0.50% and SG 0.25% supplementation concentration as optimum dose for any study involving dosing of these two Ayurvedic Rasayana.

**Conclusion** In summary, KG and SG exhibit dose-dependent effects on life-history traits in *Drosophila melanogaster* . While development remains unaffected, higher concentrations negatively impact longevity and reproductive output. Lower concentrations do not significantly affect these traits under laboratory conditions, providing a defined experimental dosage range for future studies. KG at 0.50% and SG at 0.25% do not significantly alter development, survival or overall fecundity under standard laboratory conditions and therefore represent suitable working concentrations for experimental applications.

## Methods

***Drosophila* stock husbandry:** All the experiments were conducted using wild type ( *Oregon R ^+^ ) Drosophila melanogaster.* *Drosophila* stocks were maintained using standard fly medium comprising 4.6% cornmeal, 4.5% sucrose, 1.6% yeast extract, 0.7% agar, supplemented with 0.3% propionic acid, and 0.3% hydroxybenzoic acid methyl ester. All stocks were maintained at 25°C on a 12 h light/ 12 h dark cycle. For experiments the flies/eggs were selected and transferred on the food media containing the supplementation food according to the experiment setup.

To prepare supplementation food, freshly prepared standard food media was separated according to the requirement and cooled down to the 35 to 40 ^ᵒ^ C, then the desired supplementation was mixed homogeneously according to the concentration requirement and poured into the vials (2 ml per vial and 20 ml per bottle) and left to settle (as described in Singh et al., 2018). Both the supplementations were procured from the Arya Vaidya Sala, Kottakal, Kerala with the following composition:

**Brahmi Ghrita:**

**Botanical name&nbsp;&nbsp;&nbsp;&nbsp;&nbsp;&nbsp;&nbsp;&nbsp;&nbsp;&nbsp;&nbsp;&nbsp;&nbsp;&nbsp;&nbsp;&nbsp;&nbsp;&nbsp;&nbsp;&nbsp;&nbsp;&nbsp;&nbsp;&nbsp;&nbsp;&nbsp;&nbsp;&nbsp;&nbsp;&nbsp;&nbsp;&nbsp;&nbsp; Part&nbsp;&nbsp;&nbsp;&nbsp;&nbsp;&nbsp;&nbsp;&nbsp;&nbsp;&nbsp;&nbsp;&nbsp;&nbsp;&nbsp;&nbsp; Form&nbsp; &nbsp;&nbsp;&nbsp;&nbsp;&nbsp;&nbsp;&nbsp;&nbsp;&nbsp;&nbsp;&nbsp; Qty. (per 10g)**

*Ghee&nbsp;&nbsp;&nbsp;&nbsp;&nbsp;&nbsp;&nbsp;&nbsp;&nbsp;&nbsp;&nbsp;&nbsp;&nbsp;&nbsp;&nbsp;&nbsp;&nbsp;&nbsp;&nbsp;&nbsp;&nbsp;&nbsp;&nbsp;&nbsp;&nbsp;&nbsp;&nbsp;&nbsp;&nbsp;&nbsp;&nbsp;&nbsp;&nbsp;&nbsp;&nbsp;&nbsp;&nbsp;&nbsp;&nbsp;&nbsp;&nbsp;&nbsp;&nbsp;&nbsp;&nbsp;&nbsp;&nbsp;&nbsp;&nbsp;&nbsp;&nbsp; &nbsp;&nbsp; -&nbsp;&nbsp;&nbsp;&nbsp;&nbsp;&nbsp;&nbsp;&nbsp;&nbsp;&nbsp;&nbsp;&nbsp;&nbsp;&nbsp;&nbsp;&nbsp;&nbsp;&nbsp;&nbsp;* As it is &nbsp;&nbsp;&nbsp;&nbsp;&nbsp;&nbsp;&nbsp;&nbsp;&nbsp;&nbsp;&nbsp; 12.723 ml

*Terminalia chebula&nbsp;&nbsp;&nbsp;&nbsp;&nbsp;&nbsp;&nbsp;&nbsp;&nbsp;&nbsp;&nbsp;&nbsp;&nbsp;&nbsp;&nbsp;&nbsp;&nbsp;&nbsp;&nbsp;&nbsp;&nbsp;&nbsp;&nbsp;&nbsp;&nbsp;&nbsp;&nbsp;&nbsp;* Fruit&nbsp;&nbsp;&nbsp;&nbsp;&nbsp;&nbsp;&nbsp;&nbsp;&nbsp;&nbsp;&nbsp;&nbsp;&nbsp;&nbsp;&nbsp; Paste&nbsp;&nbsp;&nbsp; &nbsp;&nbsp;&nbsp;&nbsp;&nbsp;&nbsp;&nbsp;&nbsp;&nbsp;&nbsp;&nbsp; 0.092 g

*Phyllanthus emblica&nbsp;&nbsp;&nbsp;&nbsp;&nbsp;&nbsp;&nbsp;&nbsp;&nbsp;&nbsp;&nbsp;&nbsp;&nbsp;&nbsp;&nbsp;&nbsp;&nbsp;&nbsp;&nbsp;&nbsp;&nbsp;&nbsp;&nbsp;&nbsp;&nbsp;&nbsp;* Fruit&nbsp;&nbsp;&nbsp;&nbsp;&nbsp;&nbsp;&nbsp;&nbsp;&nbsp;&nbsp;&nbsp;&nbsp;&nbsp;&nbsp;&nbsp; Paste&nbsp;&nbsp;&nbsp; &nbsp;&nbsp;&nbsp;&nbsp;&nbsp;&nbsp;&nbsp;&nbsp;&nbsp;&nbsp;&nbsp; 0.092 g

*Terminalia bellirica&nbsp;&nbsp;&nbsp;&nbsp;&nbsp;&nbsp;&nbsp;&nbsp;&nbsp;&nbsp;&nbsp;&nbsp;&nbsp;&nbsp;&nbsp;&nbsp;&nbsp;&nbsp;&nbsp;&nbsp;&nbsp;&nbsp;&nbsp;&nbsp;&nbsp;&nbsp;&nbsp;* Fruit&nbsp;&nbsp;&nbsp;&nbsp;&nbsp;&nbsp;&nbsp;&nbsp;&nbsp;&nbsp;&nbsp;&nbsp;&nbsp;&nbsp;&nbsp; Paste&nbsp;&nbsp;&nbsp; &nbsp;&nbsp;&nbsp;&nbsp;&nbsp;&nbsp;&nbsp;&nbsp;&nbsp;&nbsp;&nbsp; 0.092 g

*Citrullus colocynthis&nbsp;&nbsp;&nbsp;&nbsp;&nbsp;&nbsp;&nbsp;&nbsp;&nbsp;&nbsp;&nbsp;&nbsp;&nbsp;&nbsp;&nbsp;&nbsp;&nbsp;&nbsp;&nbsp;&nbsp;&nbsp;&nbsp;&nbsp;&nbsp;&nbsp;* Plant&nbsp;&nbsp;&nbsp;&nbsp;&nbsp;&nbsp;&nbsp;&nbsp;&nbsp;&nbsp;&nbsp;&nbsp;&nbsp;&nbsp;&nbsp; Paste&nbsp;&nbsp;&nbsp; &nbsp;&nbsp;&nbsp;&nbsp;&nbsp;&nbsp;&nbsp;&nbsp;&nbsp;&nbsp;&nbsp; 0.092 g

*Elettaria cardamomum&nbsp;&nbsp;&nbsp;&nbsp;&nbsp;&nbsp;&nbsp;&nbsp;&nbsp;&nbsp;&nbsp;&nbsp;&nbsp;&nbsp;&nbsp;&nbsp;&nbsp;&nbsp;&nbsp;&nbsp;&nbsp;&nbsp;* Seed&nbsp;&nbsp;&nbsp;&nbsp;&nbsp;&nbsp;&nbsp;&nbsp;&nbsp;&nbsp;&nbsp;&nbsp;&nbsp;&nbsp;&nbsp; Paste&nbsp;&nbsp;&nbsp; &nbsp;&nbsp;&nbsp;&nbsp;&nbsp;&nbsp;&nbsp;&nbsp;&nbsp;&nbsp;&nbsp; 0.092 g

*Cedrus deodara&nbsp;&nbsp;&nbsp;&nbsp;&nbsp;&nbsp;&nbsp;&nbsp;&nbsp;&nbsp;&nbsp;&nbsp;&nbsp;&nbsp;&nbsp;&nbsp;&nbsp;&nbsp;&nbsp;&nbsp;&nbsp;&nbsp;&nbsp;&nbsp;&nbsp;&nbsp;&nbsp;&nbsp;&nbsp;&nbsp;&nbsp;&nbsp;&nbsp;* Plant&nbsp;&nbsp;&nbsp;&nbsp;&nbsp;&nbsp;&nbsp;&nbsp;&nbsp;&nbsp;&nbsp;&nbsp;&nbsp;&nbsp;&nbsp; Paste&nbsp;&nbsp;&nbsp; &nbsp;&nbsp;&nbsp;&nbsp;&nbsp;&nbsp;&nbsp;&nbsp;&nbsp;&nbsp;&nbsp; 0.092 g

*Prunus avium&nbsp;&nbsp;&nbsp;&nbsp;&nbsp;&nbsp;&nbsp;&nbsp;&nbsp;&nbsp;&nbsp;&nbsp;&nbsp;&nbsp;&nbsp;&nbsp;&nbsp;&nbsp;&nbsp;&nbsp;&nbsp;&nbsp;&nbsp;&nbsp;&nbsp;&nbsp;&nbsp;&nbsp;&nbsp;&nbsp;&nbsp;&nbsp;&nbsp;&nbsp;&nbsp;&nbsp;&nbsp;* Flower&nbsp;&nbsp;&nbsp;&nbsp;&nbsp;&nbsp;&nbsp;&nbsp;&nbsp;&nbsp;&nbsp;&nbsp; Paste&nbsp;&nbsp;&nbsp; &nbsp;&nbsp;&nbsp;&nbsp;&nbsp;&nbsp;&nbsp;&nbsp;&nbsp;&nbsp;&nbsp; 0.092 g

*Hemidesmus indicus&nbsp;&nbsp;&nbsp;&nbsp;&nbsp;&nbsp;&nbsp;&nbsp;&nbsp;&nbsp;&nbsp;&nbsp;&nbsp;&nbsp;&nbsp;&nbsp;&nbsp;&nbsp;&nbsp;&nbsp;&nbsp;&nbsp;&nbsp;&nbsp;&nbsp;&nbsp;* Root&nbsp;&nbsp;&nbsp;&nbsp;&nbsp;&nbsp;&nbsp;&nbsp;&nbsp;&nbsp;&nbsp;&nbsp;&nbsp;&nbsp;&nbsp; Paste&nbsp;&nbsp;&nbsp; &nbsp;&nbsp;&nbsp;&nbsp;&nbsp;&nbsp;&nbsp;&nbsp;&nbsp;&nbsp;&nbsp; 0.092 g

*Ichnocarpus frutescens&nbsp;&nbsp;&nbsp;&nbsp;&nbsp;&nbsp;&nbsp;&nbsp;&nbsp;&nbsp;&nbsp;&nbsp;&nbsp;&nbsp;&nbsp;&nbsp;&nbsp;&nbsp;&nbsp;&nbsp;&nbsp;&nbsp;* Root&nbsp;&nbsp;&nbsp;&nbsp;&nbsp;&nbsp;&nbsp;&nbsp;&nbsp;&nbsp;&nbsp;&nbsp;&nbsp;&nbsp;&nbsp; Paste&nbsp;&nbsp;&nbsp; &nbsp;&nbsp;&nbsp;&nbsp;&nbsp;&nbsp;&nbsp;&nbsp;&nbsp;&nbsp;&nbsp; 0.092 g

*Curcuma longa&nbsp;&nbsp;&nbsp;&nbsp;&nbsp;&nbsp;&nbsp;&nbsp;&nbsp;&nbsp;&nbsp;&nbsp;&nbsp;&nbsp;&nbsp;&nbsp;&nbsp;&nbsp;&nbsp;&nbsp;&nbsp;&nbsp;&nbsp;&nbsp;&nbsp;&nbsp;&nbsp;&nbsp;&nbsp;&nbsp;&nbsp;&nbsp;&nbsp;&nbsp;* Rhizome&nbsp;&nbsp;&nbsp;&nbsp;&nbsp;&nbsp;&nbsp;&nbsp; Paste&nbsp;&nbsp;&nbsp; &nbsp;&nbsp;&nbsp;&nbsp;&nbsp;&nbsp;&nbsp;&nbsp;&nbsp;&nbsp;&nbsp; 0.092 g

*Berberis aristata&nbsp;&nbsp;&nbsp;&nbsp;&nbsp;&nbsp;&nbsp;&nbsp;&nbsp;&nbsp;&nbsp;&nbsp;&nbsp;&nbsp;&nbsp;&nbsp;&nbsp;&nbsp;&nbsp;&nbsp;&nbsp;&nbsp;&nbsp;&nbsp;&nbsp;&nbsp;&nbsp;&nbsp;&nbsp;&nbsp;&nbsp;&nbsp;* Root&nbsp;&nbsp;&nbsp;&nbsp;&nbsp;&nbsp;&nbsp;&nbsp;&nbsp;&nbsp;&nbsp;&nbsp;&nbsp;&nbsp;&nbsp; Paste&nbsp;&nbsp;&nbsp; &nbsp;&nbsp;&nbsp;&nbsp;&nbsp;&nbsp;&nbsp;&nbsp;&nbsp;&nbsp;&nbsp; 0.092 g

*Desmodium gangeticum&nbsp;&nbsp;&nbsp;&nbsp;&nbsp;&nbsp;&nbsp;&nbsp;&nbsp;&nbsp;&nbsp;&nbsp;&nbsp;&nbsp;&nbsp;&nbsp;&nbsp;&nbsp;&nbsp;&nbsp;* Root&nbsp;&nbsp;&nbsp;&nbsp;&nbsp;&nbsp;&nbsp;&nbsp;&nbsp;&nbsp;&nbsp;&nbsp;&nbsp;&nbsp;&nbsp; Paste&nbsp;&nbsp;&nbsp; &nbsp;&nbsp;&nbsp;&nbsp;&nbsp;&nbsp;&nbsp;&nbsp;&nbsp;&nbsp;&nbsp; 0.092 g

*Pseudarthria viscida* &nbsp;&nbsp;&nbsp;&nbsp;&nbsp;&nbsp;&nbsp;&nbsp;&nbsp;&nbsp;&nbsp;&nbsp;&nbsp;&nbsp;&nbsp;&nbsp;&nbsp;&nbsp;&nbsp;&nbsp;&nbsp;&nbsp;&nbsp;&nbsp;&nbsp; Root&nbsp;&nbsp;&nbsp;&nbsp;&nbsp;&nbsp;&nbsp;&nbsp;&nbsp;&nbsp;&nbsp;&nbsp;&nbsp;&nbsp;&nbsp; Paste&nbsp;&nbsp;&nbsp; &nbsp;&nbsp;&nbsp;&nbsp;&nbsp;&nbsp;&nbsp;&nbsp;&nbsp;&nbsp;&nbsp; 0.092 g

*Callicarpa macrophylla&nbsp;&nbsp;&nbsp;&nbsp;&nbsp;&nbsp;&nbsp;&nbsp;&nbsp;&nbsp;&nbsp;&nbsp;&nbsp;&nbsp;&nbsp;&nbsp;&nbsp;&nbsp;&nbsp;&nbsp;&nbsp;* Flower&nbsp;&nbsp;&nbsp;&nbsp;&nbsp;&nbsp;&nbsp;&nbsp;&nbsp;&nbsp;&nbsp;&nbsp; Paste&nbsp;&nbsp;&nbsp; &nbsp;&nbsp;&nbsp;&nbsp;&nbsp;&nbsp;&nbsp;&nbsp;&nbsp;&nbsp;&nbsp; 0.092 g

*Valeriana jatamansi&nbsp;&nbsp;&nbsp;&nbsp;&nbsp;&nbsp;&nbsp;&nbsp;&nbsp;&nbsp;&nbsp;&nbsp;&nbsp;&nbsp;&nbsp;&nbsp;&nbsp;&nbsp;&nbsp;&nbsp;&nbsp;&nbsp;&nbsp;&nbsp;&nbsp;&nbsp;&nbsp;* Rhizome&nbsp;&nbsp;&nbsp;&nbsp;&nbsp;&nbsp;&nbsp;&nbsp; Paste&nbsp;&nbsp;&nbsp; &nbsp;&nbsp;&nbsp;&nbsp;&nbsp;&nbsp;&nbsp;&nbsp;&nbsp;&nbsp;&nbsp; 0.092 g

*Solanum anguivi&nbsp;&nbsp;&nbsp;&nbsp;&nbsp;&nbsp;&nbsp;&nbsp;&nbsp;&nbsp;&nbsp;&nbsp;&nbsp;&nbsp;&nbsp;&nbsp;&nbsp;&nbsp;&nbsp;&nbsp;&nbsp;&nbsp;&nbsp;&nbsp;&nbsp;&nbsp;&nbsp;&nbsp;&nbsp;&nbsp;&nbsp;&nbsp;* Root&nbsp;&nbsp;&nbsp;&nbsp;&nbsp;&nbsp;&nbsp;&nbsp;&nbsp;&nbsp;&nbsp;&nbsp;&nbsp;&nbsp;&nbsp; Paste&nbsp;&nbsp;&nbsp; &nbsp;&nbsp;&nbsp;&nbsp;&nbsp;&nbsp;&nbsp;&nbsp;&nbsp;&nbsp;&nbsp; 0.092 g

*Saussurea costus&nbsp;&nbsp;&nbsp;&nbsp;&nbsp;&nbsp;&nbsp;&nbsp;&nbsp;&nbsp;&nbsp;&nbsp;&nbsp;&nbsp;&nbsp;&nbsp;&nbsp;&nbsp;&nbsp;&nbsp;&nbsp;&nbsp;&nbsp;&nbsp;&nbsp;&nbsp;&nbsp;&nbsp;&nbsp;&nbsp;&nbsp;&nbsp;* Root&nbsp;&nbsp;&nbsp;&nbsp;&nbsp;&nbsp;&nbsp;&nbsp;&nbsp;&nbsp;&nbsp;&nbsp;&nbsp;&nbsp;&nbsp; Paste&nbsp;&nbsp;&nbsp; &nbsp;&nbsp;&nbsp;&nbsp;&nbsp;&nbsp;&nbsp;&nbsp;&nbsp;&nbsp;&nbsp; 0.092 g

*Rubia cordifolia* &nbsp;&nbsp;&nbsp;&nbsp;&nbsp;&nbsp;&nbsp;&nbsp;&nbsp;&nbsp;&nbsp;&nbsp;&nbsp;&nbsp;&nbsp;&nbsp;&nbsp;&nbsp;&nbsp;&nbsp;&nbsp;&nbsp;&nbsp;&nbsp;&nbsp;&nbsp;&nbsp;&nbsp;&nbsp;&nbsp;&nbsp;&nbsp;&nbsp; Root&nbsp;&nbsp;&nbsp;&nbsp;&nbsp;&nbsp;&nbsp;&nbsp;&nbsp;&nbsp;&nbsp;&nbsp;&nbsp;&nbsp;&nbsp; Paste&nbsp;&nbsp;&nbsp; &nbsp;&nbsp;&nbsp;&nbsp;&nbsp;&nbsp;&nbsp;&nbsp;&nbsp;&nbsp;&nbsp; 0.092 g

*Mesua ferrea* &nbsp;&nbsp;&nbsp;&nbsp;&nbsp;&nbsp;&nbsp;&nbsp;&nbsp;&nbsp;&nbsp;&nbsp;&nbsp;&nbsp;&nbsp;&nbsp;&nbsp;&nbsp;&nbsp;&nbsp;&nbsp;&nbsp;&nbsp;&nbsp;&nbsp;&nbsp;&nbsp;&nbsp;&nbsp;&nbsp;&nbsp;&nbsp;&nbsp;&nbsp;&nbsp;&nbsp;&nbsp;&nbsp; Flower&nbsp;&nbsp;&nbsp;&nbsp;&nbsp;&nbsp;&nbsp;&nbsp;&nbsp;&nbsp;&nbsp;&nbsp; Paste&nbsp;&nbsp;&nbsp; &nbsp;&nbsp;&nbsp;&nbsp;&nbsp;&nbsp;&nbsp;&nbsp;&nbsp;&nbsp;&nbsp; 0.092 g

*Punica granatum* &nbsp;&nbsp;&nbsp;&nbsp;&nbsp;&nbsp;&nbsp;&nbsp;&nbsp;&nbsp;&nbsp;&nbsp;&nbsp;&nbsp;&nbsp;&nbsp;&nbsp;&nbsp;&nbsp;&nbsp;&nbsp;&nbsp;&nbsp;&nbsp;&nbsp;&nbsp;&nbsp;&nbsp;&nbsp;&nbsp;&nbsp; Seed&nbsp;&nbsp;&nbsp;&nbsp;&nbsp;&nbsp;&nbsp;&nbsp;&nbsp;&nbsp;&nbsp;&nbsp;&nbsp;&nbsp;&nbsp; Paste&nbsp;&nbsp;&nbsp; &nbsp;&nbsp;&nbsp;&nbsp;&nbsp;&nbsp;&nbsp;&nbsp;&nbsp;&nbsp;&nbsp; 0.092 g

*Embelis ribes* &nbsp;&nbsp;&nbsp;&nbsp;&nbsp;&nbsp;&nbsp;&nbsp;&nbsp;&nbsp;&nbsp;&nbsp;&nbsp;&nbsp;&nbsp;&nbsp;&nbsp;&nbsp;&nbsp;&nbsp;&nbsp;&nbsp;&nbsp;&nbsp;&nbsp;&nbsp;&nbsp;&nbsp;&nbsp;&nbsp;&nbsp;&nbsp;&nbsp;&nbsp;&nbsp;&nbsp;&nbsp; Seed&nbsp;&nbsp;&nbsp;&nbsp;&nbsp;&nbsp;&nbsp;&nbsp;&nbsp;&nbsp;&nbsp;&nbsp;&nbsp;&nbsp;&nbsp; Paste&nbsp;&nbsp;&nbsp; &nbsp;&nbsp;&nbsp;&nbsp;&nbsp;&nbsp;&nbsp;&nbsp;&nbsp;&nbsp;&nbsp; 0.092 g

*Abies spectabilis* &nbsp;&nbsp;&nbsp;&nbsp;&nbsp;&nbsp;&nbsp;&nbsp;&nbsp;&nbsp;&nbsp;&nbsp;&nbsp;&nbsp;&nbsp;&nbsp;&nbsp;&nbsp;&nbsp;&nbsp;&nbsp;&nbsp;&nbsp;&nbsp;&nbsp;&nbsp;&nbsp;&nbsp;&nbsp;&nbsp;&nbsp;&nbsp; Leaf&nbsp;&nbsp;&nbsp;&nbsp;&nbsp;&nbsp;&nbsp;&nbsp;&nbsp;&nbsp;&nbsp;&nbsp;&nbsp;&nbsp;&nbsp;&nbsp; Paste&nbsp;&nbsp;&nbsp; &nbsp;&nbsp;&nbsp;&nbsp;&nbsp;&nbsp;&nbsp;&nbsp;&nbsp;&nbsp;&nbsp; 0.092 g

*Elettaria cardamomum&nbsp;&nbsp;&nbsp;&nbsp;&nbsp;&nbsp;&nbsp;&nbsp;&nbsp;&nbsp;&nbsp;&nbsp;&nbsp;&nbsp;&nbsp;&nbsp;&nbsp;&nbsp;&nbsp;&nbsp;&nbsp;&nbsp;* Seed&nbsp;&nbsp;&nbsp;&nbsp;&nbsp;&nbsp;&nbsp;&nbsp;&nbsp;&nbsp;&nbsp;&nbsp;&nbsp;&nbsp;&nbsp; Paste&nbsp;&nbsp;&nbsp; &nbsp;&nbsp;&nbsp;&nbsp;&nbsp;&nbsp;&nbsp;&nbsp;&nbsp;&nbsp;&nbsp; 0.092 g

*Jasminum grandiflorum* &nbsp;&nbsp;&nbsp;&nbsp;&nbsp;&nbsp;&nbsp;&nbsp;&nbsp;&nbsp;&nbsp;&nbsp;&nbsp;&nbsp;&nbsp;&nbsp;&nbsp;&nbsp;&nbsp;&nbsp;&nbsp; Flower bud&nbsp;&nbsp;&nbsp;&nbsp;&nbsp; Paste&nbsp;&nbsp;&nbsp; &nbsp;&nbsp;&nbsp;&nbsp;&nbsp;&nbsp;&nbsp;&nbsp;&nbsp;&nbsp;&nbsp; 0.092 g

*Kaempferia rotunda* &nbsp;&nbsp;&nbsp;&nbsp;&nbsp;&nbsp;&nbsp;&nbsp;&nbsp;&nbsp;&nbsp;&nbsp;&nbsp;&nbsp;&nbsp;&nbsp;&nbsp;&nbsp;&nbsp;&nbsp;&nbsp;&nbsp;&nbsp;&nbsp;&nbsp;&nbsp; Rhizome&nbsp;&nbsp;&nbsp;&nbsp;&nbsp;&nbsp;&nbsp;&nbsp; Paste&nbsp;&nbsp;&nbsp; &nbsp;&nbsp;&nbsp;&nbsp;&nbsp;&nbsp;&nbsp;&nbsp;&nbsp;&nbsp;&nbsp; 0.092 g

*Baliospermum montanum&nbsp;&nbsp;&nbsp;&nbsp;&nbsp;&nbsp;&nbsp;&nbsp;&nbsp;&nbsp;&nbsp;&nbsp;&nbsp;&nbsp;&nbsp;&nbsp;&nbsp;&nbsp;* Root&nbsp;&nbsp;&nbsp;&nbsp;&nbsp;&nbsp;&nbsp;&nbsp;&nbsp;&nbsp;&nbsp;&nbsp;&nbsp;&nbsp;&nbsp; Paste&nbsp;&nbsp;&nbsp; &nbsp;&nbsp;&nbsp;&nbsp;&nbsp;&nbsp;&nbsp;&nbsp;&nbsp;&nbsp;&nbsp; 0.092 g

*Prunus cerasoides* &nbsp;&nbsp;&nbsp;&nbsp;&nbsp;&nbsp;&nbsp;&nbsp;&nbsp;&nbsp;&nbsp;&nbsp;&nbsp;&nbsp;&nbsp;&nbsp;&nbsp;&nbsp;&nbsp;&nbsp;&nbsp;&nbsp;&nbsp;&nbsp;&nbsp;&nbsp;&nbsp;&nbsp;&nbsp; Stem Bark&nbsp;&nbsp;&nbsp;&nbsp;&nbsp;&nbsp; Paste&nbsp;&nbsp;&nbsp; &nbsp;&nbsp;&nbsp;&nbsp;&nbsp;&nbsp;&nbsp;&nbsp;&nbsp;&nbsp;&nbsp; 0.092 g

*Santalum album&nbsp;&nbsp;&nbsp;&nbsp;&nbsp;&nbsp;&nbsp;&nbsp;&nbsp;&nbsp;&nbsp;&nbsp;&nbsp;&nbsp;&nbsp;&nbsp;&nbsp;&nbsp;&nbsp;&nbsp;&nbsp;&nbsp;&nbsp;&nbsp;&nbsp;&nbsp;&nbsp;&nbsp;&nbsp;&nbsp;&nbsp;&nbsp;&nbsp;* Plant&nbsp;&nbsp;&nbsp;&nbsp;&nbsp;&nbsp;&nbsp;&nbsp;&nbsp;&nbsp;&nbsp;&nbsp;&nbsp;&nbsp;&nbsp; Paste&nbsp;&nbsp;&nbsp; &nbsp;&nbsp;&nbsp;&nbsp;&nbsp;&nbsp;&nbsp;&nbsp;&nbsp;&nbsp;&nbsp; 0.092 g

**Sarasvata Ghrita:**

**Botanical name&nbsp;&nbsp;&nbsp;&nbsp;&nbsp;&nbsp;&nbsp;&nbsp;&nbsp;&nbsp;&nbsp;&nbsp;&nbsp;&nbsp;&nbsp;&nbsp;&nbsp;&nbsp;&nbsp;&nbsp;&nbsp;&nbsp;&nbsp;&nbsp;&nbsp;&nbsp;&nbsp;&nbsp;&nbsp;&nbsp;&nbsp;&nbsp;&nbsp; Part&nbsp;&nbsp;&nbsp;&nbsp;&nbsp;&nbsp;&nbsp;&nbsp;&nbsp;&nbsp;&nbsp;&nbsp;&nbsp;&nbsp;&nbsp; Form&nbsp; &nbsp;&nbsp;&nbsp;&nbsp;&nbsp;&nbsp;&nbsp;&nbsp;&nbsp;&nbsp;&nbsp; Qty. (per 10g)**

*Ghee&nbsp;&nbsp;&nbsp;&nbsp;&nbsp;&nbsp;&nbsp;&nbsp;&nbsp;&nbsp;&nbsp;&nbsp;&nbsp;&nbsp;&nbsp;&nbsp;&nbsp;&nbsp;&nbsp;&nbsp;&nbsp;&nbsp;&nbsp;&nbsp;&nbsp;&nbsp;&nbsp;&nbsp;&nbsp;&nbsp;&nbsp;&nbsp;&nbsp;&nbsp;&nbsp;&nbsp;&nbsp;&nbsp;&nbsp;&nbsp;&nbsp;&nbsp;&nbsp;&nbsp;&nbsp;&nbsp;&nbsp;&nbsp;&nbsp;&nbsp;&nbsp; &nbsp;&nbsp;&nbsp; -&nbsp;&nbsp;&nbsp;&nbsp;&nbsp;&nbsp;&nbsp;&nbsp;&nbsp;&nbsp;&nbsp;&nbsp;&nbsp;&nbsp;&nbsp;&nbsp;&nbsp;&nbsp;* As it is &nbsp;&nbsp;&nbsp;&nbsp;&nbsp;&nbsp;&nbsp;&nbsp;&nbsp;&nbsp;&nbsp; 11.826 ml

*Terminalia chebula&nbsp;&nbsp;&nbsp;&nbsp;&nbsp;&nbsp;&nbsp;&nbsp;&nbsp;&nbsp;&nbsp;&nbsp;&nbsp;&nbsp;&nbsp;&nbsp;&nbsp;&nbsp;&nbsp;&nbsp;&nbsp;&nbsp;&nbsp;&nbsp;&nbsp;&nbsp;&nbsp;&nbsp;* Fruit&nbsp;&nbsp;&nbsp;&nbsp;&nbsp;&nbsp;&nbsp;&nbsp;&nbsp;&nbsp;&nbsp;&nbsp;&nbsp;&nbsp;&nbsp; Paste&nbsp;&nbsp;&nbsp; &nbsp;&nbsp;&nbsp;&nbsp;&nbsp;&nbsp;&nbsp;&nbsp;&nbsp;&nbsp;&nbsp; 0.174 g

*Zingiber officinale&nbsp;&nbsp;&nbsp;&nbsp;&nbsp;&nbsp;&nbsp;&nbsp;&nbsp;&nbsp;&nbsp;&nbsp;&nbsp;&nbsp;&nbsp;&nbsp;&nbsp;&nbsp;&nbsp;&nbsp;&nbsp;&nbsp;&nbsp;&nbsp;&nbsp;&nbsp;&nbsp;&nbsp;&nbsp;* Rhizome&nbsp;&nbsp;&nbsp;&nbsp;&nbsp;&nbsp;&nbsp;&nbsp; Paste&nbsp;&nbsp;&nbsp; &nbsp;&nbsp;&nbsp;&nbsp;&nbsp;&nbsp;&nbsp;&nbsp;&nbsp;&nbsp;&nbsp; 0.174 g

*Piper longum&nbsp;&nbsp;&nbsp;&nbsp;&nbsp;&nbsp;&nbsp;&nbsp;&nbsp;&nbsp;&nbsp;&nbsp;&nbsp;&nbsp;&nbsp;&nbsp;&nbsp;&nbsp;&nbsp;&nbsp;&nbsp;&nbsp;&nbsp;&nbsp;&nbsp;&nbsp;&nbsp;&nbsp;&nbsp;&nbsp;&nbsp;&nbsp;&nbsp;&nbsp;&nbsp;&nbsp;&nbsp;* Fruit&nbsp;&nbsp;&nbsp;&nbsp;&nbsp;&nbsp;&nbsp;&nbsp;&nbsp;&nbsp;&nbsp;&nbsp;&nbsp;&nbsp;&nbsp; Paste&nbsp;&nbsp;&nbsp; &nbsp;&nbsp;&nbsp;&nbsp;&nbsp;&nbsp;&nbsp;&nbsp;&nbsp;&nbsp;&nbsp; 0.174 g

*Piper nigrum&nbsp;&nbsp;&nbsp;&nbsp;&nbsp;&nbsp;&nbsp;&nbsp;&nbsp;&nbsp;&nbsp;&nbsp;&nbsp;&nbsp;&nbsp;&nbsp;&nbsp;&nbsp;&nbsp;&nbsp;&nbsp;&nbsp;&nbsp;&nbsp;&nbsp;&nbsp;&nbsp;&nbsp;&nbsp;&nbsp;&nbsp;&nbsp;&nbsp;&nbsp;&nbsp;&nbsp;&nbsp;&nbsp;* Fruit&nbsp;&nbsp;&nbsp;&nbsp;&nbsp;&nbsp;&nbsp;&nbsp;&nbsp;&nbsp;&nbsp;&nbsp;&nbsp;&nbsp;&nbsp; Paste&nbsp;&nbsp;&nbsp; &nbsp;&nbsp;&nbsp;&nbsp;&nbsp;&nbsp;&nbsp;&nbsp;&nbsp;&nbsp;&nbsp; 0.174 g

*Cyclea peltata&nbsp;&nbsp;&nbsp;&nbsp;&nbsp;&nbsp;&nbsp;&nbsp;&nbsp;&nbsp;&nbsp;&nbsp;&nbsp;&nbsp;&nbsp;&nbsp;&nbsp;&nbsp;&nbsp;&nbsp;&nbsp;&nbsp;&nbsp;&nbsp;&nbsp;&nbsp;&nbsp;&nbsp;&nbsp;&nbsp;&nbsp;&nbsp;&nbsp;&nbsp;&nbsp;&nbsp;* Root&nbsp;&nbsp;&nbsp;&nbsp;&nbsp;&nbsp;&nbsp;&nbsp;&nbsp;&nbsp;&nbsp;&nbsp;&nbsp;&nbsp;&nbsp; Paste&nbsp;&nbsp;&nbsp; &nbsp;&nbsp;&nbsp;&nbsp;&nbsp;&nbsp;&nbsp;&nbsp;&nbsp;&nbsp;&nbsp; 0.174 g

*Acorus calamus* &nbsp;&nbsp;&nbsp;&nbsp;&nbsp;&nbsp;&nbsp;&nbsp;&nbsp;&nbsp;&nbsp;&nbsp;&nbsp;&nbsp;&nbsp;&nbsp;&nbsp;&nbsp;&nbsp;&nbsp;&nbsp;&nbsp;&nbsp;&nbsp;&nbsp;&nbsp;&nbsp;&nbsp;&nbsp;&nbsp;&nbsp;&nbsp;&nbsp; Rhizome&nbsp;&nbsp;&nbsp;&nbsp;&nbsp;&nbsp;&nbsp;&nbsp; Paste&nbsp;&nbsp;&nbsp; &nbsp;&nbsp;&nbsp;&nbsp;&nbsp;&nbsp;&nbsp;&nbsp;&nbsp;&nbsp;&nbsp; 0.174 g

*Moringa oleifera&nbsp;&nbsp;&nbsp;&nbsp;&nbsp;&nbsp;&nbsp;&nbsp;&nbsp;&nbsp;&nbsp;&nbsp;&nbsp;&nbsp;&nbsp;&nbsp;&nbsp;&nbsp;&nbsp;&nbsp;&nbsp;&nbsp;&nbsp;&nbsp;&nbsp;&nbsp;&nbsp;&nbsp;&nbsp;&nbsp;&nbsp;* Root&nbsp;&nbsp;&nbsp;&nbsp;&nbsp;&nbsp;&nbsp;&nbsp;&nbsp;&nbsp;&nbsp;&nbsp;&nbsp;&nbsp;&nbsp; Paste&nbsp;&nbsp;&nbsp; &nbsp;&nbsp;&nbsp;&nbsp;&nbsp;&nbsp;&nbsp;&nbsp;&nbsp;&nbsp;&nbsp; 0.174 g

*Rock salt&nbsp;&nbsp;&nbsp;&nbsp;&nbsp;&nbsp;&nbsp;&nbsp;&nbsp;&nbsp;&nbsp;&nbsp;&nbsp;&nbsp;&nbsp;&nbsp;&nbsp;&nbsp;&nbsp;&nbsp;&nbsp;&nbsp;&nbsp;&nbsp;&nbsp;&nbsp;&nbsp;&nbsp;&nbsp;&nbsp;&nbsp;&nbsp;&nbsp;&nbsp;&nbsp;&nbsp;&nbsp;&nbsp;&nbsp;&nbsp;&nbsp;&nbsp;&nbsp;&nbsp; &nbsp;&nbsp;&nbsp;* - *&nbsp;&nbsp;&nbsp;&nbsp;&nbsp;&nbsp;&nbsp;&nbsp;&nbsp;&nbsp;&nbsp;&nbsp;&nbsp;&nbsp;&nbsp;&nbsp;&nbsp;&nbsp;* Paste&nbsp;&nbsp;&nbsp; &nbsp;&nbsp;&nbsp;&nbsp;&nbsp;&nbsp;&nbsp;&nbsp;&nbsp;&nbsp;&nbsp; 0.174 g

*Goat’s milk&nbsp;&nbsp;&nbsp;&nbsp;&nbsp;&nbsp;&nbsp;&nbsp;&nbsp;&nbsp;&nbsp;&nbsp;&nbsp;&nbsp;&nbsp;&nbsp;&nbsp;&nbsp;&nbsp;&nbsp;&nbsp;&nbsp;&nbsp;&nbsp;&nbsp;&nbsp;&nbsp;&nbsp;&nbsp;&nbsp;&nbsp;&nbsp;&nbsp;&nbsp;&nbsp;&nbsp;&nbsp;&nbsp;&nbsp;&nbsp;&nbsp; &nbsp;&nbsp;&nbsp; -&nbsp;&nbsp;&nbsp;&nbsp; &nbsp;&nbsp;&nbsp;&nbsp;&nbsp;&nbsp;&nbsp;&nbsp;&nbsp;&nbsp;&nbsp;&nbsp;&nbsp;* as it is &nbsp;&nbsp;&nbsp;&nbsp;&nbsp;&nbsp;&nbsp;&nbsp;&nbsp;&nbsp;&nbsp; 11.130 ml

*&nbsp;*

**Developmental Assay:** Newly hatched *Drosophila* were fed on the supplementation food of different concentration and their developmental time was recorded for each supplementation and concentration. (as described in Ackermann et al., 2001)

**Longevity Assay:** First instar larvae eclosed within one hour of hatching were collected and fed on desired concentration of the supplementation/normal food (control food and formulated foods were taken from the same batch of the larvae to keep variation minimum). Flies were transferred to fresh vials (without anaesthesia etherizing) every alternate day until all flies are dead and no of flies alive are recorded each time. (as described in Linford et al., 2013)

**Fecundity Assay:** Male and female flies are separated within one hour of eclosion under ether anaesthesia and kept separately for three days in separate vials after that mating was set in 2*2 factorial design (2 males and 2 females) for control as well as different supplementation food feeding groups. Flies were transferred to fresh vial each day and the number of eggs laid were counted and noted every day until 45 days. (as described in Singh et al., 2018)

**Statistical analysis:** All the statistical analysis were done using graph-pad (prism 8.4.2 version). Kaplan–Meier survival analysis followed by log-rank (Mantel–Cox), one way ANOVA, and two ANOVA was performed for statistical analysis where appropriate.

**Food color/dye feeding experiment:** Bromophenol blue dye was mixed homogeneously with the food (both NF and SG/KG supplementation). IInd instar larvae were separated from the food vial from standard culture (both NF and supplementation group) media, washed and transferred in a petri plate containing dye mixed food media. Larvae were left to feed for 30-45 minutes on the dye containing food. After feeding, larvae were washed with 1x PBS and observed under stereo-binocular.
